# The Dutchess County Resolution Regarding State Censorship

**Published:** 1912-02

**Authors:** 


### BUFFALO MEDICAL JOURNAL

### The Dutchess County Resolution Regarding State Censorship.

The Medical Society of the County of Dutchess, is again asking other county societies to memoralize the State Society, with the ultimate object of placing the censorship of medical ethics in the hands of the latter, instead of the county societies, as at present. The reason is the difficulty of securing efficient control in counties of comparatively sparse population.

With the general principle of securing efficient control of medical practice, the BUFFALO MEDICAL JOURNAL is in hearty sympathy. As to the exact machinery employed, we are indifferent, provided that it accomplishes the desired result. We feel, however, that county societies of comparatively large membership, especially those in which the majority of members and the majority of offending practitioners are found in cities, are justified in insisting on the established precedent of home rule. When a county society, on mature reflexion, is convinced that it cannot properly deal with the problem, provision should be made for its transfer to the State Society. Yet, it may be worth considering whether the same factors urged for this transfer, would not tend to prevent efficient action by the central body, unless by a return to the former system of a permanent, limited State membership and by a rather elaborate and expensive official organization.

